# TaSCA, an Agile Survey on Chemosensory Impairments for Self-Monitoring of COVID-19 Patients: A Pilot Study

**DOI:** 10.3389/fneur.2021.633574

**Published:** 2021-02-24

**Authors:** Carla Mucignat-Caretta, Patrizia Bisiacchi, Gian Luigi Marcazzan, Arianna Calistri, Cristina Parolin, Angelo Antonini

**Affiliations:** ^1^Department of Molecular Medicine, University of Padova, Padua, Italy; ^2^Department of General Psychology, University of Padova, Padua, Italy; ^3^Padova Neuroscience Center, Padua, Italy; ^4^CREA—Council for Agricultural Research and Economics, Bologna, Italy; ^5^Department of Neuroscience, University of Padova, Padua, Italy

**Keywords:** COVID-19, olfaction, taste, chemesthesis, trigeminus, self-assessment, chemical senses, recovery

## Abstract

**Background/Objective:** During the COVID-19 pandemic, smell and taste disorders emerged as key non-respiratory symptoms. Due to widespread presence of the disease and to difficult objective testing of positive persons, the use of short surveys became mandatory. Most of the existing resources are focused on smell, very few on taste or trigeminal chemosensation called chemesthesis. However, it is possible that the three submodalities are affected differently by COVID-19.

**Methods:** We prepared a short survey (TaSCA) that can be administered at the telephone or through online resources to explore chemosensation. It is composed of 11 items on olfaction, taste, and chemesthesis, in order to discriminate the three modalities. We avoided abstract terms, and the use of semiquantitative scales because older patients may be less engaged. Statistical handling included descriptive statistics, Pearson's chi-squared test and cluster analysis.

**Results:** The survey was completed by 83 persons (60 females and 23 males), which reported diagnosis of COVID-19 by clinical (*n* = 7) or molecular (*n* = 18) means, the others being non-COVID subjects. Cluster analysis depicted the existence of two groups, one containing mostly asymptomatic and one mostly symptomatic subjects. All swab-positive persons fell within this second group. Only one item, related to trigeminal temperature perception, did not discriminate between the two groups.

**Conclusions:** These preliminary results indicate that TaSCA may be used to easily track chemosensory symptoms related to COVID-19 in an agile way, giving a picture of three different chemosensory modalities.

## Introduction

After the first weeks of SARS-CoV2 spreading (1) and the worldwide dissemination of COVID-19 pandemic, it became clear that the virus is able to affect different organs, while giving the most dismal outcomes in the case of respiratory tract infection (2). In addition to severe respiratory symptoms, rather non-specific signs were reported as initial manifestation of infection, like fever, myalgia, and headache^1^. However, other symptoms emerged as associated to the infection, including the loss of smell termed anosmia (3–5), whose sudden onset appeared as a typical feature of the COVID-19 disease (6, 7). Actually, other viruses may affect the ability to smell, however in these cases the loss of olfactory function is smoother and associated to various degree of nasal symptoms, like running nose (8), while in the case of COVID-19, the loss of smell may appear also in the absence of any other symptom, often in a sudden and dramatic way. The consequences of olfactory loss, whatever the cause, may be life-threatening, by reducing the awareness of potentially dangerous stimuli like gas or rotten food (9).

While the loss of smell is the most apparent sign of chemosensory involvement and can be regarded as a predictor of COVID-19 (10–14) also taste loss (ageusia) may be present, and in some cases the trigeminal chemical sense (15) called chemesthesis is involved (14, 16).

During the initial phase of the pandemic, we followed some mildly symptomatic COVID-19 patients experiencing symptoms related to the chemical senses. However, most of the existing survey at that time were focused on olfaction, and none put together olfaction, taste and chemesthesis. We observed that patients could not easily discriminate between the three different chemosensory modalities, which are sensitive to different stimuli and use a variety of transduction pathways and central connections to reach specific sensory areas in the brain. Hence, we felt compelled to create an agile tool to collect information on these three chemosensory modalities, that could be used either online or as direct or telephone interview, to allow the widest collection of data and reach even persons in remote areas.

The need for a tool easy to use and manage, that allows patients to respond and follow the evolution of their symptoms, prompted us to create a very short online survey on the three chemosensory modalities, focusing on items which are part of everyday life for most people. We referred to sensory experiences related to specific objects, instead of using more generic terms (like “smell”) and tried to discriminate between different odorant sources, in case the subject has no relevant experience: for example, one person in isolation may not have a direct contact with perfumes, yet may still retrieve some soap to smell. We also avoided the use of semi-structured scales, since these may be difficult to be adopted by older persons. Taste-Smell-Chemesthesis Agile (TaSCA) survey is presented here along with the responses collected online from April 30 to the end of May 2020, from asymptomatic, non-affected persons and COVID-19 positive patients. The aim of the present work is to show that this tool may discriminate between persons showing chemosensory systems involvement or not, and may serve as a proof-of-principle for the use of TaSCA in conditions in which direct testing is prevented.

## Methods

The survey was created to collect data for an observational, prospective single center study aimed at determining medium/long term consequences of COVID-19 on neurological status (NEUROCOVID, Ethical Committee Prot. 056881). Data herein presented were collected online in May 2020, during the first COVID-19 outbreak, by soliciting patients and non-affected persons to participate. No exclusion criteria were set, since we aimed at establishing differences between groups of patients and healthy subjects and explore the viability of TaSCA as a tool for COVID-19 patients.

The questionnaire is composed of three sheets implemented in Google Forms. One introductory sheet is entitled “COVID-19 and taste and smell disturbances” and shows an introductory statement. It presents one mandatory question, on the willingness to participate, and 3 more questions (name or nickname, gender, and age). The following sheet is the actual survey. The subject should answer to 12 questions. The first 11 questions take in consideration the sensory experience in the last days, with respect to the usual sensitivity. One of four responses is possible: (1) No change in the last period (I sense as always), (2) Moderate change (I sense less than usual),^3^ Loss of function (I don't sense those items), (3) Don't know/don't remember, in the case the person did not had the chance to sense the item in the last period, because of physical constraints. The questions explore smell (questions 1, 3, 5), nasal and oral chemesthesis (questions 2, 4, 10, 11), and taste (questions 6, 7, 8, 9). The last question refers to the presence of symptoms related to COVID-19, with the possibility to add some notes. The third and last sheet is salutation and thanks. The questionnaire in the original language is available at the link:

https://docs.google.com/forms/d/1yvCBD8QBWTv1dOahp YWVhMRfS-8WaESCx80jhDIBW_Q/viewform?ts=5ea2c7 76&edit_requested=true

**Figure 1:**
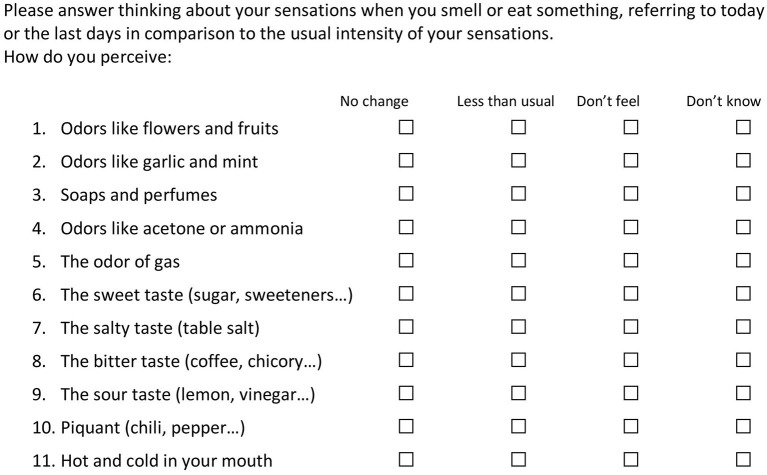
TaSCA questions on taste, smell, and chemesthesis impairment.

The printout is presented in the Supplementary Material 1, while the English translation is shown in Figure 1.

### Data Analysis

Data were analyzed using IBM SPSS Statistics v26 (RRID:SCR_019096) to produce descriptive statistics, Pearson's chi-squared analysis and cluster analysis. Descriptive statistics were calculated for each item.

As a first step, we asked whether the items were able to indicate a difference among persons experiencing a change in sensitivity and COVID-19 status. Hence, the 4 types of responses were coded as 0 (no change in sensitivity, answer 1) or 1 (there was a change in the last period, answers 2 and 3). Concerning answer 4, we manually coded it as 0 if all the other responses indicated no change.

The hierarchical agglomerative algorithm according to Ward's method was applied for clustering (17), since this warrants robust results (18), using squared Euclidean distance for assessing similarity and Pearson's chi-square to test for difference between the clusters for each item. Significance level was set at *p* < 0.0045 after Bonferroni correction for multiple comparisons.

## Results

Eighty-three persons completed the survey: 60 females and 23 males. 56 were asymptomatic, 7 had a COVID-19 clinical diagnosis based on symptoms (because during the first pandemic, access to naso-pharyngeal swab was restricted to patients with severe symptoms), 18 had a SARS-CoV2 positive nasopharyngeal swab and 2 self-reported sudden olfactory loss but when the swab test become available, it turned out to be negative. Age ranged from 19 to 73 years old (mean ± SD: 35.98 ± 16.61). All subjects presented valid data: percentages of response for each item are presented in Table 1.

**Table 1:** Percentage (%) of response and number (N) of persons reporting a modification in their sensitivity.

	**0—Don't Know**	**1—Don't Feel**	**2—Moderate Change**	**3—No Change**	**No. of persons reporting a change**
ITEM 1	1.2%	19.3	9.6	69.9	24
Flowers	*N* = 1	16	8	58	
ITEM 2	12.0	15.7	8.4	63.9	20
Mint	10	13	7	53	
ITEM 3	0	19.3	8.4	72.3	23
Perfumes	0	16	7	60	
ITEM 4	13.3	13.3	3.6	69.9	14
Acetone	11	11	3	58	
ITEM 5	26.5	8.4	4.8	60.2	11
Gas	22	7	4	50	
ITEM 6	1.2	7.2	10.8	80.7	15
Sweet taste	1	6	9	67	
ITEM 7	2.4	9.6	12.0	75.9	18
Salty taste	2	8	10	63	
ITEM 8	8.4	7.2	12.0	71.1	17^*^
Bitter taste	7	6	10	59	
ITEM 9	6.0	6.0	8.4	79.5	12
Sour taste	5	5	7	66	
ITEM 10	21.7	4.8	4.8	68.7	8
Piquant	18	4	4	57	
ITEM 11	2.4	0	3.6	94.0	3
Temperature	2	0	3	78	

* *One person reported hypersensitivity*.

In the open question, 42 subjects (50.6%) reported no symptoms related to COVID-19, 27 (32.5%) declared some symptoms and 14 (16.9) answered “I don't know.”

Several items had 5 cases or less for the responses 0-1-2 (Table 1): 6 for response 0, 5 for response 1, and 4 for response 2. Due to the low frequencies, the responses were categorized in 2 classes namely, 0: No change compared to previous sensitivity, 1: Change compared to previous sensitivity (see Table 1, last column).

**Figure 2:**
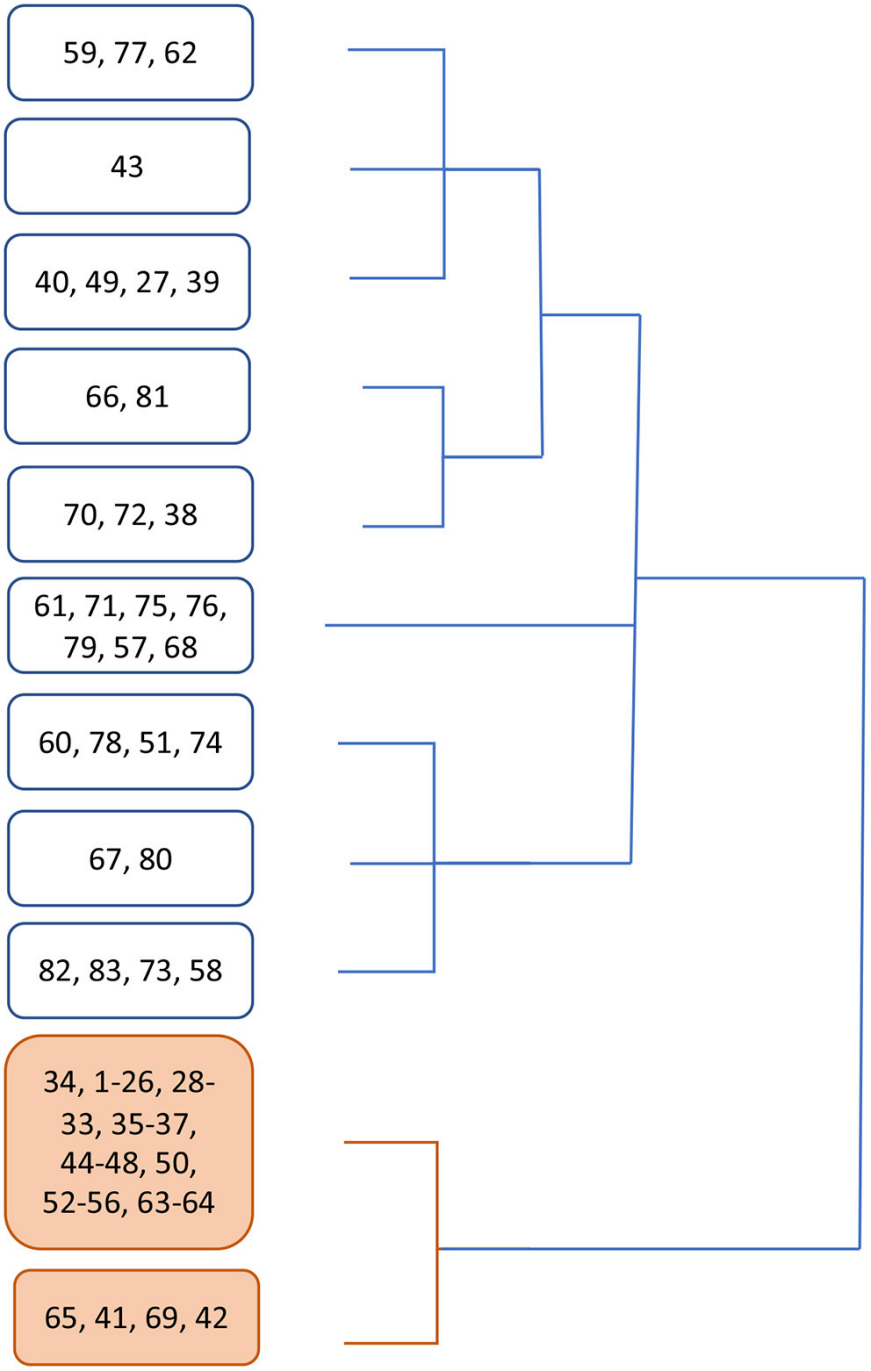
Dendrogram showing clustering emerged from cluster analysis. In white boxes, the branch containing mostly symptomatic patients, in orange the branch containing no swab-positive subject.

Cluster analysis showed that two main groups emerged from data, one composed of 53 subjects (63.8%) and the other of 30 subjects (36.2%). The relative dendrogram is shown in Figure 2.

Apparently, group 1 mostly collects asymptomatic subjects (92.5%), while 73% persons in group 2 received a diagnosis of COVID-19 (Supplementary Material 2).

Supplementary Material 3 reports the distribution of subjects in the two groups emerged from the cluster analysis with respect to the absence or presence of modification in sensitivity: for items 1 to 10, the percentage of subjects reporting a change in sensitivity is significantly different in the two groups. Only for item 11, related to temperature perception, the reported change in sensitivity is not significantly different between the two groups (Supplementary Material 3).

Supplementary Material 4 reports the raw data for the cluster analysis.

**Figure 3:**
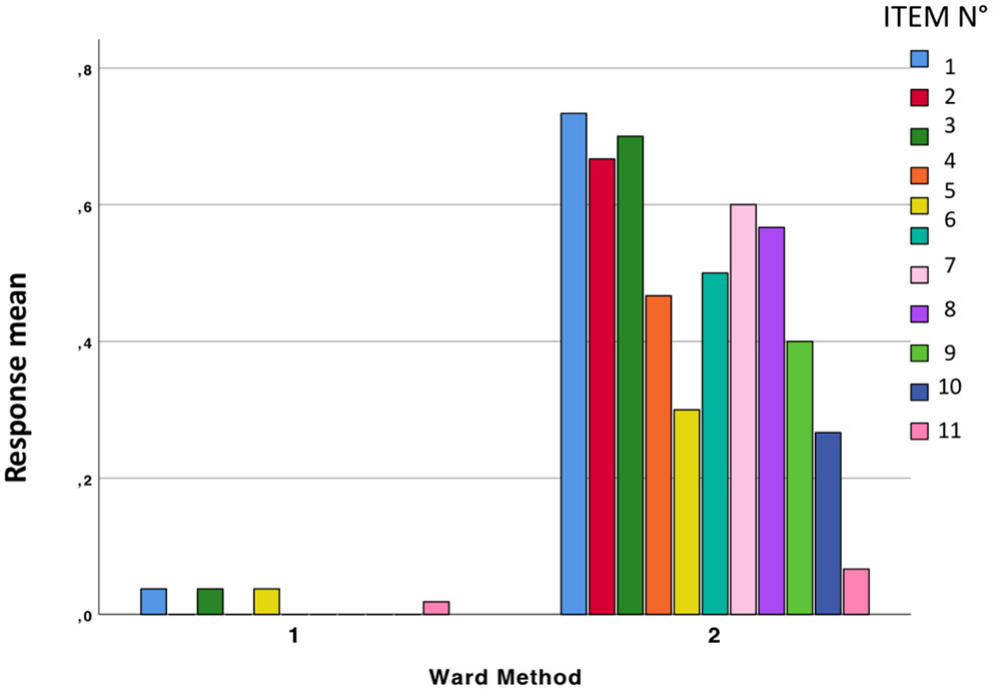
The two groups emerging from cluster analysis present a different response pattern.

The mean responses in the two groups are shown in Figure 3.

Concerning the last item, on the presence of COVID-19-related symptoms, 3 responses were analyzed: (1) Yes, (2) No, (3) I don't know. The two groups emerged from cluster analysis significantly differed between each other (Pearsons' chi-squared = 42.344, df = 2, *p* < 0.0001). In group 1, 71.7% (38 subjects) reported no symptoms, 7.5% (*n* = 4) reported symptoms and 20.8% (*n* = 11) did not know, while in group 2 13.3% (*n* = 4) did not report symptoms, 76.7% reported symptoms (*n* = 23), and 10% (*n* = 3) did not know.

## Discussion

Often regarded as an ancillary sense, olfaction has a great role in our everyday life for its involvement in lifeguarding processes (e.g., avoiding dangerous chemicals) as well in food evaluation.

The COVID-19 pandemic brought to the attention of clinicians an astonishing number of persons infected with mild symptoms, the most intriguing being a sudden and complete loss of olfaction (6), which may last for a variable period of time, often accompanied by taste and chemesthesis impairment (14). While anecdotical self-reports are intriguing, to pose a diagnosis and follow the course of the disease, it is necessary to develop instruments that allow a reliable quantification of the functional impairment.

In the case of chemical senses, two main objective tests have been developed and used over the years in the clinic, namely the Sniffin' Sticks (19) and the University of Pennsylvania Smell Inventory Test (UPSIT) (20), while others are being developed. Their use is mandatory to have an objective evaluation of the impairment, since the subjective report is often misleading (21). However, they both require the direct testing of the patient, which may be difficult or impossible in the case of COVID-19 infected persons. On the contrary, collecting data from patients may be of paramount importance to follow the disease. Sadly, the utility of self-reporting about chemical sensitivity has been repeatedly questioned (22).

Many questionnaires are available to test olfactory function and some of them are widely used (23–25). However, in the case of COVID-19 disease some caution should be warranted. First, most of the currently available surveys are focused on either smell or taste and almost none take into consideration the trigeminal chemical sensitivity. However, one of the most intriguing feature of COVID-19-related chemical senses impairment is the involvement of multiple chemosensory modalities in the disease, as well as their sudden loss of function. Hence, the development of surveys specifically targeting all three chemical sensitivity is long needed. Another feature of COVID-19 disease is the wide range of age of affected persons, yet the diagnostic tools should be easily accessible to most of them: hence, we avoided the use of visual-analog scales, which may be more sensitive than other descriptors but less flexible in terms of accessibility.

Other tools have been developed, including the 40-items GCCR multi-lingual online questionnaire (14), however this requires the access to the web and a certain degree of confidence in using online surveys, which may prevent older persons to access it. Moreover, given the worldwide diffusion of the pandemic, it may be useful to create agile instruments that are easily administered using different tools, including self-administration, direct interviews or over the phone, besides online presentation. Lastly, while most available tools may be complete and hence rather long, we focused the questions on the three chemical modalities to have a glimpse of their relative degree of impairment. By keeping the questions tied to real objects that could be sensed and avoiding abstract terms, we tried to grasp the sensory experience in a more faithful way.

Present data show that mostly asymptomatic patients cluster together, while most patients experiencing chemosensory impairment, with particular involvement of olfaction, cluster together, suggesting that TaSCA survey may be a valuable tool in case of impossibility to administer more objective tests. It may also help in collecting critical information on the objective sensitivity in a fast and easy way.

The present work is limited to the initial collections of a case-series to test whether it could discriminate between symptomatic and non-symptomatic patients. Since it relied on the voluntary participation of subjects, we did not attempt to refine sampling, to stay closer to the real-life use: hence, the sex balance is skewed toward females, probably because females are more compliant and willing to take part in preliminary testing. Similarly, we did not take any action on age balance, leaving this for future investigations.

Some initial speculations are possible on the present data. Whether items concerning olfaction are the most discriminative indexes, compared to taste and trigeminal will be explored in future work. Also, with a larger sample it will be possible to fully assess the psychometric and statistical properties of the questionnaire, including for example case-control approaches and cutoff point determination. Interestingly, item 11 on thermal sensitivity does not appear to discriminate between the 2 groups that emerged from cluster analysis. This suggests that the trigeminus nerve may be differentially affected in its different sensory components. Chemical sensitivity in trigeminal afferents involves receptors which are also temperature-gated (26, 27), but cold receptors also exist (28–30), and functional specializations have been reported for trigeminal receptors (31). It is worth to include item 11 because it may discriminate among persons experiencing true chemosensory deficit from deceitful answers.

While obtained in a limited number of subjects, these data show that TaSCA is a short survey available in the same form online, on paper or for oral interview that may be used to screen chemosensory deficits in COVID-19 patients. It remains to be determined its possible use in other conditions where these sensory systems may be involved.

In COVID-19 patients which are still positive, isolated at home or when direct objective testing is not feasible or recommendable, TaSCA could be easily administered. It is fast, yet complete in exploring all three chemosensory modalities and could be used also with patients still presenting annoying symptoms, which may prevent them from extensive sessions of online or live testing.

Possibly, it could be useful in the future for repeated self-checking with substances commonly available in most houses, given the necessity of long-term monitoring for possible adverse outcomes (32).

## Data Availability Statement

The original contributions presented in the study are included in the article/Supplementary Material, further inquiries can be directed to the corresponding author/s.

## Ethics Statement

The studies involving human participants were reviewed and approved by Comitato Etico per la Sperimentazione Clinica della Provincia di Padova (affiliation: Padova province). The patients/participants provided their written informed consent to participate in this study.

## Author Contributions

CM-C conceived the questionnaire. CM-C and PB analyzed the data. GM prepared the online material. CM-C, GM, AC, and CP participated in data collection. AA provided financial support. All authors drafted the manuscript.

## Conflict of Interest

The authors declare that the research was conducted in the absence of any commercial or financial relationships that could be construed as a potential conflict of interest.

## Footnotes

^1^https://www.cdc.gov/coronavirus/2019-ncov/symptoms-testing/symptoms.html (accessed November 23, 2020).

## References

[1] Zhu N Zhang D Wang W Li X Yang B Song J. A novel coronavirus from patients with pneumonia in China, 2019. N Engl J Med. (2020) 382:727–33. 10.1056/NEJMoa2001017 31978945 PMC7092803

[2] Varga Z Flammer AJ Steiger P Haberecker M Andermatt R Zinkernagel AS. Endothelial cell infection and endotheliitis in COVID-19. Lancet. (2020) 395:1417–8. 10.1016/S0140-6736(20)30937-5 32325026 PMC7172722

[3] Lechien JR Chiesa-Estomba CM De Siati DR Horoi M Le Bon SD Rodriguez A. Olfactory and gustatory dysfunctions as a clinical presentation of mild-to-moderate forms of the coronavirus disease (COVID-19): a multicenter European study. Eur Arch Otorhinolaryngol. (2020) 277:2251–61. 10.1007/s00405-020-05965-1 32253535 PMC7134551

[4] Moein ST Hashemian SM Mansourafshar B Khorram-Tousi A Tabarsi P Doty RL. Smell dysfunction: a biomarker for COVID-19. Int Forum Allergy Rhinol. (2020) 10:944-950. 10.1002/alr.22587 32301284 PMC7262123

[5] Hornuss D Lange B Schröter N. Anosmia in COVID-19 patients. Clin Microbiol Infect. (2020) 26:1426–7. 10.1016/j.cmi.2020.05.017 32447049 PMC7242197

[6] Gane SB Kelly C Hopkins C. Isolated sudden onset anosmia in COVID-19 infection. a novel syndrome? Rhinology. (2020) 58:299–301. 10.4193/Rhin20.114 32240279

[7] Whitcroft KL Hummel T. Olfactory dysfunction in COVID-19: diagnosis and management. JAMA. (2020) 323:2512–4. 10.1001/jama.2020.8391 32432682

[8] Seiden AM. Postviral olfactory loss. Otolaryngol Clin North Am. (2004) 37:1159–66. 10.1016/j.otc.2004.06.007 15563908

[9] Rebholz H Braun RJ Ladage D Knoll W Kleber C Hassel AW. Loss of olfactory function-early indicator for Covid-19, other viral infections and neurodegenerative disorders. Front Neurol. (2020) 11:569333. 10.3389/fneur.2020.569333 33193009 PMC7649754

[10] Rocke J Hopkins C Philpott C Kumar N. Is loss of sense of smell a diagnostic marker in COVID-19: a systematic review and meta-analysis. Clin Otolaryngol. (2020) 1:13620. 10.1111/coa.13620 32741085 PMC7436734

[11] Menni C Valdes AM Freidin MB Sudre CH Nguyen LH Drew DA. Real-time tracking of self-reported symptoms to predict potential COVID-19. Nat Med. (2020) 26:1037–40. 10.1038/s41591-020-0916-2 32393804 PMC7751267

[12] Seden N Yigit E Yigit Ö Kaygisiz I. Objective evaluation of odor loss in COVID-19 and other suspected cases. Am J Otolaryngol. (2020) 42:102761. 10.1016/j.amjoto.2020.102761 33080550 PMC7556258

[13] Parma V Ohla K Veldhuizen MG Niv MY Kelly CE Bakke AJ. More than smell – COVID-19 is associated with severe impairment of smell, taste, and chemesthesis. Chem Senses. (2020) 45:609–22. 10.1093/chemse/bjaa041 32564071 PMC7337664

[14] Gerkin RC Ohla K Veldhuizen MG Joseph PV Kelly CE Bakke AJ. Recent smell loss is the best predictor of COVID-19 among individuals with recent respiratory symptoms. Chem Senses. (2020) 25:bjaa081. 10.1093/chemse/bjaa081 33367502 PMC7799216

[15] Viana F. Chemosensory properties of the trigeminal system. ACS Chem Neurosci. (2011) 2:38–50. 10.1021/cn100102c 22778855 PMC3369707

[16] Cooper KW Brann DH Farruggia MC Bhutani S Pellegrino R Tsukahara T. COVID-19 and the chemical senses: supporting players take center stage. Neuron. (2020) 107:219–33. 10.1016/j.neuron.2020.06.032 32640192 PMC7328585

[17] Ward JHJ. Hierarchical grouping to optimize an objective function. J Am Stat Ass. (1963) 58:236–44.

[18] Milligan GW. A review of Monte Carlo tests of cluster analysis. Multivar Behav Res. (1981) 16:379–407. 26815599 10.1207/s15327906mbr1603_7

[19] Hummel T Sekinger B Wolf SR. ‘Sniffin' Sticks’: olfactory performance assessed by the combined testing of odor identification, odor discrimination and olfactory threshold. Chem Senses. (1997) 22:39–52. 9056084 10.1093/chemse/22.1.39

[20] Doty RL Shaman P Kimmelman CP Dann MS. University of Pennsylvania smell identification test: A rapid quantitative olfactory function test for the clinic. Laryngoscope. (1984) 94:176–8. 6694486 10.1288/00005537-198402000-00004

[21] Hummel T Whitcroft KL Andrews P Altundag A Cinghi C Costanzo RM. Position paper on olfactory dysfunction. Rhinology. (2016) 56:1–30. 10.4193/Rhin16.248 28623665

[22] Lötsch J Hummel T. Clinical usefulness of self-rated olfactory performance-a data science-based assessment of 6000 patients. Chem Senses. (2019) 44:357–64. 10.1093/chemse/bjz029 31077277

[23] Zou LQ Linden L Cuevas M Metasch ML Welge-Lüssen A Hähner A. Self-reported mini olfactory questionnaire (Self-MOQ): a simple and useful measurement for the screening of olfactory dysfunction. Laryngoscope. (2020) 130:E786–E90. 10.1002/lary.28419 31747076

[24] Mullol J Alobid I Mariño-Sánchez F Quintó L de Haro J Bernal-Sprekelsen M. Furthering the understanding of olfaction, prevalence of loss of smell and risk factors: a population-based survey (OLFACAT study). BMJ Open. (2012) 2:e001256. 10.1136/bmjopen-2012-001256 23135536 PMC3533119

[25] Langstaff L Pradhan N Clark A Boak D Salam M Hummel T. Validation of the olfactory disorders questionnaire for English- speaking patients with olfactory disorders. Clin Otolaryngol. (2019) 44:715–28. 10.1111/coa.13351 31038840

[26] Silver WL Clapp TR Stone LM Kinnamon SC. TRPV1 receptors and nasal trigeminal chemesthesis. Chem Senses. (2006) 31:807–12. 10.1093/chemse/bjl022 16908491

[27] Simon SA Gutierrez R. Chapter 7: TRP channels at the periphery of the taste and trigeminal systems. In: Emir TLR, editor. Neurobiology of TRP Channels. Boca Raton, FL: CRC Press; Taylor & Francis; (2017). 29356478

[28] Babes A. Ion channels involved in cold detection in mammals: TRP and non-TRP mechanisms. Biophys Rev. (2009) 1:193–200. 10.1007/s12551-009-0020-9 28510025 PMC5425670

[29] Kichko TI Neuhuber W Kobal G Reeh PW. The roles of TRPV1, TRPA1 and TRPM8 channels in chemical and thermal sensitivity of the mouse oral mucosa. Eur J Neurosci. (2018) 47:201–10. 10.1111/ejn.13799 29247491

[30] Iftinca M Altier C. The cool things to know about TRPM8!. Channels. (2020) 14:413–20. 10.1080/19336950.2020.1841419 33147416 PMC7657583

[31] Lemon CH Kang Y Li J. Separate functions for responses to oral temperature in thermo-gustatory and trigeminal neurons. Chem Senses. (2016) 41:457–71. 10.1093/chemse/bjw02 26976122 PMC4910675

[32] Bougakov D Podell K Goldberg E. Multiple Neuroinvasive Pathways in COVID-19. Mol Neurobiol. (2020) 29:1–12. 10.1007/s12035-020-02152-5 PMC7523266 32990925

