# Supplementary material for: TaSCA, an Agile Survey on Chemosensory Impairments for Self-Monitoring of COVID-19 Patients: A Pilot Study
Source: Front Neurol. 2021 Feb 24;12:633574. doi: 10.3389/fneur.2021.633574 (PMC7943440; doi:10.3389/fneur.2021.633574)
Supplement: Supplementary file 1 [file Data_Sheet_1.PDF]

Supplementary Material

Supplementary Material 1.

The TaSCA survey as downloaded in the original (Italian) language.

COVID-19 e disturbi dell’olfatto e del gusto

Chiediamo la sua collaborazione, rispondendo ad un breve questionario che ci aiuti a capire quali possono essere i sintomi lievi legati a COVID-19. La compilazione richiederà pochi minuti.

*Campo obbligatorio

Acconsente a compilare il questionario? *

○ Sì

○ No

Nickname

La tua risposta

Sesso

○ M

○ F

COVID-19 e disturbi dell'olfatto e del gusto

Le chiediamo di rispondere relativamente a ciò che sente quando mangia o quando annusa qualcosa.

Si riferisca ad oggi o alle ultime giornate, rispetto alla sua sensibilità media.

1. Come sente gli odori come fiori e frutta?

- [ ] come sempre
- [ ] meno del solito
- [ ] non lo sento più
- [ ] non so/non ci ho fatto caso

2. gli odori come aglio e menta

- [ ] come sempre
- [ ] meno del solito
- [ ] non lo sento più
- [ ] non so/non ci ho fatto caso

COVID-19 e disturbi dell'olfatto e del gusto

Grazie per la partecipazione

Indietro Invia

Non inviare mai le password tramite Moduli Google.

Questi contenuti non sono creati né avallati da Google. Segnala una violazione - Termini di servizio - Norme sulla privacy

Google Moduli

Supplementary Material 2

Clustering of subjects according to symptoms

	Total no. of subjects	Group 1	Group 2
Not COVID19	56	49	7
Positive swab	18	0	18
Clinical diagnosis	7	3	4
Negative swab	2	1	1

Supplementary Material 3

Change in sensitivity in the two cluster of subjects.

SUPPLEMENTARY MATERIAL 3		% NO change in sensitivity	% changed sensitivity	Chi-squared	P
ITEM 1	Group 1	96.2	3.8	45.095	0.0001
flowers	Group 2	26.7	73.3		
ITEM 2	Group 1	100	0	46.550	0.0001
mint	Group 2	33.3	66.7		
ITEM 3	Group 1	96.2	3.8	41.943	0.0001
perfumes	Group 2	30.0	70.0		
ITEM4	Group 1	100	0	29.752	0.0001
acetone	Group 2	53.3	46.7		
ITEM 5	Group 1	96.2	3.8	11.461	0.001
gas	Group 2	70	30		
ITEM 6	Group 1	100	0	32.346	0.0001
sweet taste	Group 2	50	50		
ITEM 7	Group 1	100	0	40.606	0.0001
salty taste	Group 2	40	60		
ITEM 8	Group 1	100	0	37.769	0.0001
bitter taste	Group 2	43.3	56.7		
ITEM 9	Group 1	100	0	24.783	0.0001
sour taste	Group 2	60	40		
ITEM 10	Group 1	100	0	15.641	0.0001
piquant	Group 2	73.3	26.7		
ITEM 11	Group 1	98.1	1.9	1.256	0.262
temperature	Group 2	93.3	6.7		

Supplementary Material 4

Raw data

N	Sample	Gender	Age	ITEM 1	ITEM 2	ITEM 3	ITEM 4	ITEM 5	ITEM 6	ITEM7	ITEM 8	ITEM 9	ITEM 10	ITEM 11	SYMPTOMS	CLU2_1
1	NO SYMPTOMS	F	50	0	0	0	0	0	0	0	0	0	0	0	Don't know	1
2	NO SYMPTOMS	F	20	0	0	0	0	0	0	0	0	0	0	0	No	1
3	NO SYMPTOMS	F	53	0	0	0	0	0	0	0	0	0	0	0	No	1
4	NO SYMPTOMS	F	22	0	0	0	0	0	0	0	0	0	0	0	No	1
5	NO SYMPTOMS	F	20	0	0	0	0	0	0	0	0	0	0	0	No	1
6	NO SYMPTOMS	F	19	0	0	0	0	0	0	0	0	0	0	0	No	1
7	NO SYMPTOMS	F	20	0	0	0	0	0	0	0	0	0	0	0	Don't know	1
8	NO SYMPTOMS	F	50	0	0	0	0	0	0	0	0	0	0	0	Don't know	1
9	NO SYMPTOMS	F	21	0	0	0	0	0	0	0	0	0	0	0	No	1
10	NO SYMPTOMS	F	20	0	0	0	0	0	0	0	0	0	0	0	No	1
11	NO SYMPTOMS	F	20	0	0	0	0	0	0	0	0	0	0	0	No	1
12	NO SYMPTOMS	F	55	0	0	0	0	0	0	0	0	0	0	0	No	1
13	NO SYMPTOMS	F	22	0	0	0	0	0	0	0	0	0	0	0	No	1
14	NO SYMPTOMS	F	28	0	0	0	0	0	0	0	0	0	0	0	No	1
15	NO SYMPTOMS	F	48	0	0	0	0	0	0	0	0	0	0	0	Don't know	1
16	NO SYMPTOMS	F	23	0	0	0	0	0	0	0	0	0	0	0	No	1
17	NO SYMPTOMS	F	23	0	0	0	0	0	0	0	0	0	0	0	No	1
18	NO SYMPTOMS	F	22	0	0	0	0	0	0	0	0	0	0	0	No	1
19	NO SYMPTOMS	F	23	0	0	0	0	0	0	0	0	0	0	0	No	1
20	NO SYMPTOMS	F	22	0	0	0	0	0	0	0	0	0	0	0	Don't know	1
21	NO SYMPTOMS	F	25	0	0	0	0	0	0	0	0	0	0	0	No	1
22	NO SYMPTOMS	F	22	0	0	0	0	0	0	0	0	0	0	0	Don't know	1
23	NO SYMPTOMS	F	22	0	0	0	0	0	0	0	0	0	0	0	No	1
24	NO SYMPTOMS	F	24	0	0	0	0	0	0	0	0	0	0	0	No	1
25	NO SYMPTOMS	F	23	0	0	0	0	0	0	0	0	0	0	0	No	1
26	NO SYMPTOMS	F	54	0	0	0	0	0	0	0	0	0	0	0	No	1
27	NO SYMPTOMS	F	36	0	1	0	0	0	0	1	0	0	0	0	Don't know	2
28	NO SYMPTOMS	F	50	0	0	0	0	0	0	0	0	0	0	0	Don't know	1
29	NO SYMPTOMS	F	47	0	0	0	0	0	0	0	0	0	0	0	No	1
30	NO SYMPTOMS	F	22	0	0	0	0	0	0	0	0	0	0	0	No	1
31	NO SYMPTOMS	F	47	0	0	0	0	0	0	0	0	0	0	0	No	1
32	NO SYMPTOMS	F	20	0	0	0	0	0	0	0	0	0	0	0	No	1
33	NO SYMPTOMS	F	19	0	0	0	0	0	0	0	0	0	0	0	No	1
34	NO SYMPTOMS	F	21	0	0	0	0	0	0	0	0	0	0	1	No	1
35	NO SYMPTOMS	F	21	0	0	0	0	0	0	0	0	0	0	0	No	1
36	NO SYMPTOMS	F	23	0	0	0	0	0	0	0	0	0	0	0	No	1
37	NO SYMPTOMS	F	26	0	0	0	0	0	0	0	0	0	0	0	Don't know	1
38	NO SYMPTOMS	F	53	1	1	0	0	0	1	1	1	0	0	0	Don't know	2
39	NO SYMPTOMS	F	53	0	0	0	0	0	0	1	0	0	0	0	No	2
40	NO SYMPTOMS	F	36	0	0	0	0	0	0	1	1	0	0	0	Don't know	2
41	NO SYMPTOMS	F	26	0	0	0	0	1	0	0	0	0	0	0	Don't know	1
42	NO SYMPTOMS	F	22	1	0	0	0	0	0	0	0	0	0	0	No	1
43	NO SYMPTOMS	F	22	0	1	0	1	0	1	0	0	0	0	1	No	2
44	NO SYMPTOMS	M	73	0	0	0	0	0	0	0	0	0	0	0	No	1
45	NO SYMPTOMS	M	21	0	0	0	0	0	0	0	0	0	0	0	Don't know	1
46	NO SYMPTOMS	M	24	0	0	0	0	0	0	0	0	0	0	0	No	1
47	NO SYMPTOMS	M	53	0	0	0	0	0	0	0	0	0	0	0	No	1
48	NO SYMPTOMS	M	20	0	0	0	0	0	0	0	0	0	0	0	No	1
49	NO SYMPTOMS	M	20	0	0	0	0	0	0	1	1	0	0	0	No	2
50	NO SYMPTOMS	M	22	0	0	0	0	0	0	0	0	0	0	0	No	1
51	NO SYMPTOMS	M	43	1	0	1	1	0	0	0	0	0	0	0	No	2
52	NO SYMPTOMS	M	61	0	0	0	0	0	0	0	0	0	0	0	Don't know	1
53	NO SYMPTOMS	M	22	0	0	0	0	0	0	0	0	0	0	0	No	1
54	NO SYMPTOMS	M	67	0	0	0	0	0	0	0	0	0	0	0	No	1
55	NO SYMPTOMS	M	20	0	0	0	0	0	0	0	0	0	0	0	No	1
56	NO SYMPTOMS	M	36	0	0	0	0	0	0	0	0	0	0	0	No	1
57	POSITIVE SWAB	F	20	1	1	1	1	1	1	1	1	1	1	1	Yes	2
58	POSITIVE SWAB	F	21	1	1	1	0	1	1	0	1	0	0	0	Yes	2
59	POSITIVE SWAB	F	61	0	0	0	0	0	1	1	1	1	0	0	Yes	2
60	POSITIVE SWAB	M	68	1	1	1	1	0	0	0	1	0	0	0	Yes	2
61	POSITIVE SWAB	M	48	1	1	1	1	1	1	1	1	1	0	0	Yes	2
62	CLINICAL DIAGNC	F	36	0	0	1	0	0	1	0	0	1	0	0	Yes	2
63	CLINICAL DIAGNC	F	45	0	0	0	0	0	0	0	0	0	0	0	Yes	1
64	CLINICAL DIAGNC	F	46	0	0	0	0	0	0	0	0	0	0	0	Yes	1
65	CLINICAL DIAGNC	F	23	0	0	1	0	1	0	0	0	0	0	0	Yes	1
66	CLINICAL DIAGNC	F	72	1	1	1	0	0	0	1	0	1	0	0	Yes	2
67	CLINICAL DIAGNC	M	71	1	1	1	1	1	0	0	1	0	1	0	Yes	2
68	CLINICAL DIAGNC	M	29	1	1	1	1	1	1	1	1	1	1	0	Yes	2
69	NEGATIVE SWAB	F	48	1	0	1	0	0	0	0	0	0	0	0	Yes	1
70	NEGATIVE SWAB	F	33	1	1	1	0	0	1	1	0	0	0	0	Yes	2
71	POSITIVE SWAB	F	46	1	1	1	1	1	1	1	1	1	0	0	Yes	2
72	POSITIVE SWAB	F	49	1	1	1	0	1	1	1	0	0	0	0	Yes	2
73	POSITIVE SWAB	M	61	1	1	1	0	0	0	0	1	0	1	0	Yes	2
74	POSITIVE SWAB	M	61	1	0	1	1	0	0	0	1	0	0	0	Yes	2
75	POSITIVE SWAB	F	24	1	1	1	1	0	1	1	1	1	0	0	Yes	2
76	POSITIVE SWAB	F	30	1	1	1	1	0	1	1	1	1	1	0	Yes	2
77	POSITIVE SWAB	F	61	0	0	0	0	0	1	1	1	1	0	0	Yes	2
78	POSITIVE SWAB	M	68	1	1	1	1	0	0	0	0	0	0	0	Yes	2
79	POSITIVE SWAB	F	31	1	0	1	1	0	1	1	1	1	1	0	Yes	2
80	POSITIVE SWAB	M	34	1	1	1	1	1	0	0	0	0	1	0	Yes	2
81	POSITIVE SWAB	F	38	1	0	0	0	0	0	1	0	1	1	0	Yes	2
82	POSITIVE SWAB	M	26	1	1	1	0	1	0	0	0	0	0	0	Yes	2
83	POSITIVE SWAB	M	59	1	1	1	0	0	0	0	0	0	0	0	Yes	2
